# A Replication-Competent HIV Clone Carrying GFP-Env Reveals Rapid Env Recycling at the HIV-1 T Cell Virological Synapse

**DOI:** 10.3390/v14010038

**Published:** 2021-12-25

**Authors:** Lili Wang, Alice Sandmeyer, Wolfgang Hübner, Hongru Li, Thomas Huser, Benjamin K. Chen

**Affiliations:** 1 Department of Medicine, Division of Infectious Disease, Immunology Institute, Icahn School of Medicine at Mount Sinai, New York, NY 10029, USA; liliwang582@gmail.com (L.W.); hongru.li@mssm.edu (H.L.); 2 Biomolecular Photonics, Department of Physics, University of Bielefeld, 33615 Bielefeld, Germany; asandmeyer@physik.uni-bielefeld.de (A.S.); whuebner@physik.uni-bielefeld.de (W.H.); thomas.huser@physik.uni-bielefeld.de (T.H.)

**Keywords:** HIV-1, virological synapse, Env, GFP, fluorescence recovery after photobleaching FRAP, Gag, endocytosis

## Abstract

HIV-1 infection is enhanced by cell–cell adhesions between infected and uninfected T cells called virological synapses (VS). VS are initiated by the interactions of cell-surface HIV-1 envelope glycoprotein (Env) and CD4 on target cells and act as sites of viral assembly and viral transfer between cells. To study the process that recruits and retains HIV-1 Env at the VS, a replication-competent HIV-1 clone carrying an Env-sfGFP fusion protein was designed to enable live tracking of Env within infected cells. Combined use of surface pulse-labeling of Env and fluorescence recovery after photobleaching (FRAP) studies, enabled the visualization of the targeted accumulation and sustained recycling of Env between endocytic compartments (EC) and the VS. We observed dynamic exchange of Env at the VS, while the viral structural protein, Gag, was largely immobile at the VS. The disparate exchange rates of Gag and Env at the synapse support that the trafficking and/or retention of a majority of Env towards the VS is not maintained by entrapment by a Gag lattice or immobilization by binding to CD4 on the target cell. A FRAP study of an Env endocytosis mutant showed that recycling is not required for accumulation at the VS, but is required for the rapid exchange of Env at the VS. We conclude that the mechanism of Env accumulation at the VS and incorporation into nascent particles involves continuous internalization and targeted secretion rather than irreversible interactions with the budding virus, but that this recycling is largely dispensable for VS formation and viral transfer across the VS.

## 1. Introduction

The HIV-1 envelope glycoprotein (Env) plays crucial roles as the surface glycoprotein on the virus particle, mediating virus binding, fusion and entry, as well as in initiating the formation of cell–cell adhesions that facilitate viral transmission, called virological synapses (VS) [1,2,3]. HIV-1 can infect cells through cell-free virus, or through cell-to-cell routes which involve direct transfer of virus across a VS. HIV-1 Env is the surface antigen exposed on the surface of the cell or the virus particle where it can engage its main molecular target CD4. HIV-1 is an enveloped virus that assembles and buds from the plasma membrane in a process mediated by the core structural protein Gag [4]. Trafficking through endocytic pathways enhances packaging of Env onto nascent virus particles [5,6,7]. Although Env engagement of CD4 at the cell surface is required for VS formation, the expression of Env at the cell surface renders infected cells susceptible to antibody detection and, while many antibodies against Env can block the formation of virological synapses, they are less efficient at blocking cell-to-cell infection than they are at blocking cell-free infection [8,9,10,11,12].

The biogenesis of HIV-1 Env begins at ribosomes on the rough endoplasmic reticulum (ER) where newly synthesized Env is glycosylated into precursor gp160 to form homotrimers [13]. The cleavage of gp160 occurs in the Golgi apparatus by furin or furin-like proteases and results in two non-covalently associated peptides: a cell surface glycoprotein, gp120, and a transmembrane glycoprotein, gp41 [14,15]. Env trimers travel through the secretory pathway to reach the plasma membrane, and then are quickly recycled from the cell surface [16,17,18,19,20]. This contributes to the very low number of Env glycoproteins on the cell surface. HIV-1 gp41 has a long intracytoplasmic C-terminal tail compared to other retroviruses [21,22]. A membrane-proximal tyrosine-based sorting signal YxxL in the gp41 C-terminus interacts with the AP-2 to promote the internalization of Env [23,24,25]. Env recycling from the cell surface to the endocytic recycling compartment (ERC) is a prerequisite for Env incorporation [6,7]. This internalized Env may also proceed through retrograde trafficking pathways to the Golgi prior to Env incorporation [26]. Proper incorporation of Env into viral particles also requires gp41 C-terminal sequences [27] and compatibility with complementary sequences in Gag [28,29]. The outward trafficking of Env from ERC to the virus assembly area is mediated by C-terminal tyrosine-based motif YW795 [5].

HIV-1 cell-to-cell transmission leads to the efficient transfer of virus and infection [3,10,30] and mediates resistance to neutralization [8,9,10,11,12]. Cell-to-cell transmission promotes viral diversity by supporting the co-transmission of multiple copies of HIV-1 per transmission event [31,32,33,34] and is proposed to play a role in escape from immune responses or may promote the evolution of drug resistance in settings of suboptimal therapy [9,35,36]. The HIV-1 VS is an example of polarized viral transmission, where the assembly and release of Env and Gag are directed toward the receiving target cell, which internalizes the virus through an endocytic pathway [37,38]. At the VS, HIV-1 Gag, Env and CD4 localize to the site of cell–cell contact in an actin-dependent manner [3]. Recruitment of Gag and Env protein and their transfer through VS occurs in a dynamic process following cell-adhesion [39,40]. Env-CD4 interaction is required for VS formation. Blocking the interaction of Env and CD4 with antibodies inhibits VS formation [10]. During the formation of a VS, Env is observed to accumulate at the VS; however, the mechanisms of enrichment of Env at the VS are not well characterized. The extent to which Env diffuses laterally within the membrane to the VS from surface pools or may be concentrated by a secretory pathway that targets the VS is unclear.

The fusion of proteins with the green fluorescent protein enables live tracking of the protein within the cell [41]. However, the relatively large size of GFP and its derivatives (30 kD) requires careful consideration of the site of insertion to maintain the function of the protein of interest. Prior Env–GFP fusions have been expressed outside of the full proviral context or required complementation of WT Env to support viral replication [39,42]. To preserve Env function, Nakane et al. found that a strategy of insertion of GFP into the fourth and fifth variable loops, can retain many Env functions while creating a fluorescent Env-GFP fusion protein [42]. These studies screened GFP insertions using a panel of 16 positions within variable loops with respect to cell–surface expression, cell–cell fusion activity and entry competence of pseudotyped particles and found that insertion of GFP can be compatible with virus incorporation and viral membrane fusion [42].

Short peptide motifs in the Env cytoplasmic tail (CT) can control surface Env levels, direct incorporation of Env into viral particles, and can impact the conformation of the surface [42] domain of Env, which can further modulate Env fusogenic potential [43,44]. In this study, we engineered an infectious HIV-1 carrying a fluorescent-Env to observe the *de novo* expression of Env in an infected cell and track Env accumulation and turnover during VS formation. We followed the turnover rate of Env trafficking at the VS using fluorescence recovery after photobleaching (FRAP), which revealed that surface Env is constitutively recycled and the residence time at the cell surface is short lived measured in minutes, even at sites of high surface accumulation.

## 2. Materials and Methods

### 2.1. Cell Lines and Virus Production

The CD4+ T-cell line Jurkat CE6.1 (ATCC) and CD4+ T-cell line MT4 were maintained in RPMI 1640 with 100 U/mL penicillin, 100 U/mL streptomycin and 10% fetal bovine serum (FBS). Cells were maintained at concentrations of less than 10^6^/mL. Cell-free virus was produced by transfection of 293 T cells in 10 cm plates using polyjet (Signagen, Frederick, MD, USA). Media was exchanged 16 h post transfection and virus supernatants were harvested 48 h post transfection.

### 2.2. Human Primary CD4 T Cells

Primary CD4+ T cells were obtained from human peripheral blood from deidentified HIV-negative blood donors, through the New York Blood Center and CD4+ cells isolated by negative selection with a Miltenyi CD4 T cell isolation kit II (Miltenyi Biotec, Cambridge, MA, USA). Unactivated CD4+ T cells were maintained in complete RPMI medium containing 50 U/mL interleukin 2 (IL-2; HIV Reagent Program, Manassus, VA, USA). Activated primary CD4+ cells maintained by co-culture with irradiated PBMC feeder cells plus 100 U/mL IL-2 and 4 µg/mL PHA for 3 days.

### 2.3. Viruses

HIV Gag-iGFP and HIV Gag-iCherry are full-length molecular clones of HIV based on NL4-3 [45] previously designed to carry the green fluorescent protein (GFP) or mCherry protein inserted between the Gag MA and CA domains [46]. HIV constructs with fluorescent Env were constructed by inserting Superfolder green fluorescent protein (sfGFP) internally into the Env V4 or V5 domains, designated HIV Env- HIV V4.1-sfGFP, HIV Env-V4.2-sfGFP, HIV Env-V5.2-sfGFP or HIV Env-V5.3-sfGFP. The superfolder GFP is introduced by 2-step PCR with the primers shown in key resource table. These fluorescent Env genes are also inserted into the context of HIV Gag-iCherry to yield constructs carrying Gag-iCherry and Env-sfGFP in *cis*. Y712A mutant was introduced by site mutation primer shown in key resource table. HIV clone, NL-sfGI is a replication-competent molecular clone with a WT Env, that carries sf GFP in the nef position, and an internal ribosome entry site (IRES) in front of an intact nef ORF that restores strong nef expression.

### 2.4. p24 ELISA

Costar 3922 flat-bottomed, high binding plates were coated with anti-p24 capture antibody overnight (Aalto D7320; 1:200 in 0.1 M NaHCO_3_). The plates were washed twice with 1× TBST and blocked with 2% nonfat dry milk (Lab Scientific, Livingston, NJ, USA) for 1 h then washed in TBST. HIV supernatants treated with 1% Empigen (1:100 and 1:1000 in DMEM) along with titration of p24 standard are added to wells and incubated at room temperature for 2 h, then washed 4× with TBST. Alkaline phosphatase conjugated mouse anti-HIV p24 (CLINIQA, San Marcos, CA, USA) was added (1:8000 in TBST 20% sheep serum) and incubated for 1 h followed by 6 TBST washes. A total of 50 μL of Sapphire Substrate (ThermoFisher, Waltham, MA, USA) was added to each well and incubated for 20 min. Luminescence was quantitated on a Fluo Star Optima plate reader and sample values were calculated based on nonlinear regression of standard curve using Prism software (Graphpad Inc., San Diego, CA, USA).

### 2.5. Western Blot Analysis

Cells or virus were lysed with an RIPA buffer and protease inhibitor cocktail (Sigma). Protein loaded from viral lysates were normalized to p24 antigen content. Viral supernatants normalized to p24 content were spun at low speed to remove cellular debris, and virus was concentrated by ultracentrifugation by pelleting through 20% sucrose phosphate buffered saline (PBS) and resuspended to RIPA lysis buffer. Lysate equivalent of approximately 2 × 10^5^ cells per well were run on NuPage 4–12% Bis-Tris Gel (Invitrogen, Waltham, MA, USA) and transferred to Amersham Hybond-P PVDF membranes (GE Healthcare, Chicago, IL, USA). Membranes were blocked with 2% nonfat dry milk (Lab Scientific), then probed with rabbit anti-GFP serum (1:5000) or human anti-HIV serum (1:10,000) primary antibodies followed by anti-rabbit (Jackson Immunoresearch, West Grove, PA, USA) or anti-human horseradish peroxidase (Jackson Immunoresearch) conjugated secondary antibody. Detection of HRP signal was performed using Super Signal West Femto Maximum Sensitivity Substrate (Thermo Fisher Scientific, Waltham, MA, USA).

### 2.6. Infectivity TZM-bl Assay

Cell-free viruses were produced by transient transfection of 293 T cells. TZM-bl cells were plated at 2 × 10^4^ cells/well in 96-well plates and incubated at 37 °C with indicated viruses. Media was replaced after 24 h of infection and incubated for another 24 h. At 48 h post infection, media was aspirated followed by lysis in luciferase cell culture lysis reagent (Promega, Madison, WI, USA). A total of 20μL of each sample was read on Fluo Star Optima plate reader with injection of 50 μL of luciferase assay reagent (Promega).

### 2.7. Cell-to-Cell Transfer Assay

To generate virus-producing donor cells, HIV-1 proviral constructs were transfected into Jurkat cells (donor cells) using Amaxa nucleofection, as previously described (Amaxa Biosystems, Lonza, Basel, Switzerland). In brief, 5 μg of endotoxin-free HIV-1 proviral plasmids was nucleofected into 6 × 10^6^ Jurkat cells using Cell Line Nucleofector kit V, program S-18. Twenty hours after nucleofection, viable Jurkat cells were purified by centrifugation on a Ficoll-Hypaque density gradient, washed with RPMI containing 10% FBS, and recovered at 37 °C for co-culture. Unactivated primary CD4^+^ T cells (target cells) were cultured overnight in complete RPMI medium containing 50 U/mL IL-2. Donor and target cells were mixed at a ratio of approximately 1:1 and cocultured at 37 °C for 3 h before they were treated with trypsin to remove surface-attached virus and fixed. Where inhibitor Leu3a, an HIV-blocking anti-CD4 antibody (BD Biosciences, 1 μg/mL) was used, donor and target cells were preincubated separately with equal volumes of inhibitor for 30 min at 37 °C before mixing.

### 2.8. Growth Curve of HIV Env-V4.2-sfGFP

HIV-1 Env-V4.2-sfGFP virus was produced by transfecting 293T cells. 25ul of virus containing supernatant was used to spinoculate Jurkat cells in each well of 96-well plate, with or without pre-treatment of 10ul AZT and Nelfinavir respectively. 24 h post spinoculation, cell culture media was changed with fresh RPMI and without 10ul of AZT and Nelfinavir. Cells were collected every two days and processed for flow cytometry and intracellular p24 staining.

### 2.9. Fluorescence Microscopy Sample Preparation

Transfected Jurkat cells (donor cells) were mixed with primary CD4 cells (target cells) in round bottom 96-well-plates for 3–4 h as previously described. Transfer with wide bore pipette tips were used to reduce disruption of cell–cell conjugates. Co-cultured donor and target cells were transferred onto poly-l-lysine treated coverslips for 30 min at 37 °C. Cells were fixed with 4% paraformaldehyde (PFA) for 10 min at room temperature, washed twice with PBS, and mounted with anti-fade mounting medium with DAPI (Vectashield, Co#: H-1200, Vector Laboratories, Burlingame, CA, USA). For intracellular staining of Env with 2G12, transfected Jurkat cells were plated onto poly-l-lysine treated cover glass and allowed to attach for 30 min at 37 °C. The fixed cells were permeabilized with PBS containing 0.1% triton X-100 and 2% FBS for 5 min. The cells were stained with 2G12 (1:200) for 1 h followed by secondary antibody for 45 min. For surface staining of Env, the cells were directly stained at 4 °C with anti-GFP antibody (1:500) diluted in PBS with 2% FBS for 45 min, followed by a secondary antibody for 30 min, and then washed and fixed in 4% PFA or kept alive for live cell pulse-chase experiments.

### 2.10. Confocal and Live Imaging

Confocal imaging was carried out on an inverted Leica SP5 DMI laser scanning confocal microscope, using a 63× objective and analyzed using Volocity (PerkinElmer, Hopkinton, MA, USA) or ImageJ (NIH, Bethesda, MD, USA) software. Live imaging was carried out in a sealed, gas permeable microchamber slides (Ibidi Biosciences, Gräfelfing, Germany). Donor cells were mixed with target cells at a ratio of 1:2 and were loaded onto the micro-chamber pre-coated with 150 μg/mL fibronectin to provide the cells with a two- dimensional substrate for attachment and migration. The chamber was placed on a Zeiss AxioObserver Z1 inverted microscope mounted with Yokogawa CSU-X1 spinning disk scan head. Dual Hamamatsu EM-CCD C9100 digital cameras enable simultaneous imaging of up to two fluorescent channels. Phase contrast imaging and confocal green (for sfGFP) and red (for mCherry) fluorescence were acquired in a multitrack configuration to avoid cross-talk between fluorescence channels. Images were recorded at different time intervals continuously as indicated in results. Confocal images and Quicktime movies were generated from laser-scanning confocal microscope file data using using Metamorph software (Molecular Devices, San Jose, CA, USA) and Imaris (Bitplane, Zurich, Switzerland) software.

### 2.11. Fluorescence Recovery after Photobleaching (FRAP)

FRAP was performed on two systems: Zeiss LSM880 and Leica SP5 DMI. Zeiss LSM880 Airyscan microscope equipped with a 63X oil-immersion objective (NA 1.4) using the 561 nm and 488 nm laser lines. The system is adjusted to proper humidity, 5% CO_2_ and 37 °C. The FRAP experiment on LSM880 used a 4-min protocol: pre-bleach for 3 s, bleach for 1 sec at 60% laser power and recovery of fluorescence was captured for the last of the 4 min. On Leica SP5 DMI, we used a 60× oil-immersion objective (NA1.4) with 561 nm and 488 nm laser lines. There is an inherent three-step capturing protocol from the system. After 1 s bleaching, the first 100 frames were captured continuously; the second 50 frames were at 1 s/frame and the last 50 frames at 5 s/frame. A rectangular zone covering about half of the virological synapse was bleached, leaving the other half as unbleached area control and localization reference. In one case, where the virological synapse was too small to bleach a fraction of it, a nearby area was selected as unbleached area control. FRAP curve of the bleached virological synapse was determined from ROI rigidly covering the synapse button. A normal bleaching curve was determined from a different area covering most of the cytoplasm of the same cell and used for normalization of values. Fluorescence intensity over time was plotted using GraphPad Prism 9, and the data were fitted to a one-phase exponential association function to calculate recovery half-times and immobile fractions.

### 2.12. Super-Resolution Optical Microscopy of HIV-Infected T Cells

3D structured illumination microscopy of fixed T cells was performed with a commercial Deltavision OMXv4.0 BLAZE microscope (GE Healthcare, Amersham, UK) using a 60×, 1.42 NA oil immersion PlanApoN objective lens (Olympus, Tokyo, Japan) and sCMOS cameras. Env tagged with sfGFP was excited at 488 nm and the emission recorded at 504–552 nm. Gag tagged with mCherry was excited at 546 nm and the emission recorded at 600-650 nm. The plasma membrane was stained with CellMask Deep Red, excited at 649 nm and the emission recorded at 660–670 nm. The nucleus was stained with DAPI, excited at 405 nm and the emission recorded at 450–470 nm. A sequence of 15 images for each axial plane, obtained at three different angles with five phases each, was acquired. Multiple axial planes encompassing the entire cell from top to bottom were recorded at a separation of the individual axial planes of 125 nm. Super-resolved fluorescent images were reconstructed with the corresponding recorded optical transfer function (OTF) in the SoftWoRx 7.0.0 software (GE Healthcare) at a Wiener filter setting of 0.006.

## 3. Results

### 3.1. Engineering an Infectious HIV Carrying a sfGFP Insertion into the Env V4 or V5 Domains

**Figure 1:**
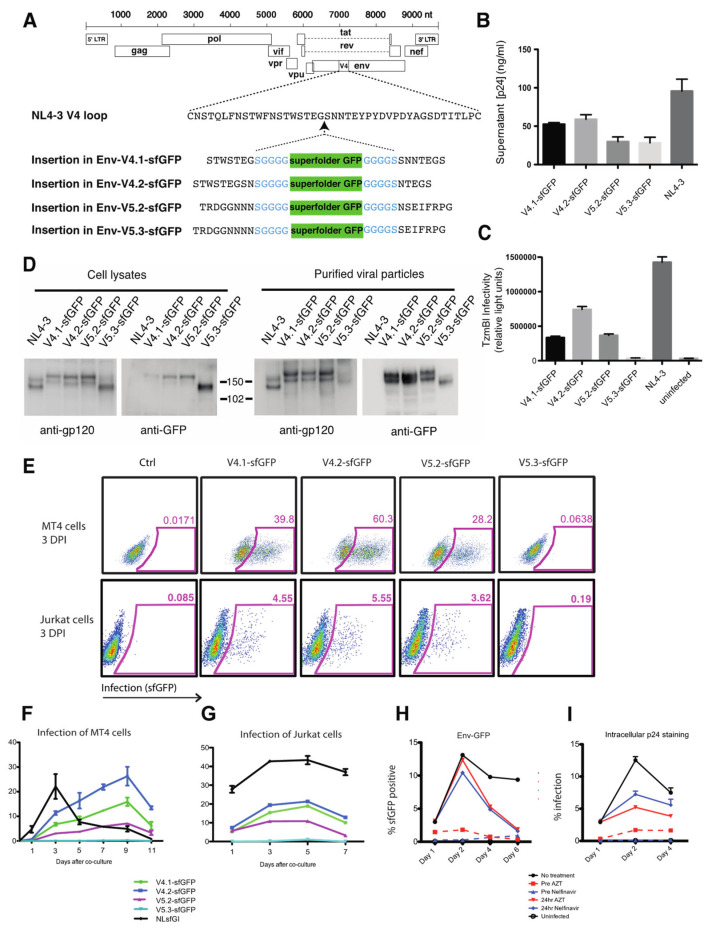
Construction of infectious HIV clones with fluorescent Env carrying sfGFP inserted into V4 or V5 domains. (**A**) sfGFP is inserted into HIV-1(NL4-3) in V4 or V5. (**B**) Virus production by fluorescent Env HIV constructs following transfection of 293 T cells measured by p24 ELISA. (**C**) Cell-free virus infectivity was tested by infection of indicator cell line, Tzm-bl. Tzm-bl cells were infected with viral supernatants containing equivalent p24 antigen. (**D**) Western blot analysis of lysates of transfected 293 T cells or of virus particles harvested from transfected cell supernatants and purified through a 20% sucrose cushion. Blots were probed with anti-gp120 or anti-GFP antibody. Viral supernatants and cell lysates were collected at 48 h post transfection. (**E**) Infection of Jurkat cells or MT4 cells with virus was assessed on day 3 post infection. (**F**) Infection of MT4 cells initiated by co-culture with HIV-nucleofected Jurkat T cells. Flow cytometry was used to monitor the fraction of MT4 cells infected over time. (**G**) Infection of Jurkat cells initiated by spinnoculation of Jurkat T cells with cell-free virus. Flow cytometry was used to monitor the fraction of Jurkat cells infected over time. (**H**) Growth curve of HIV Env V4.2-sfGFP in Jurkat cells with and without treatment of AZT and Nelfinavir prior to or 24 h after infection measured by Env-GFP flow cytometry and (**I**) intracellular p24 staining.

To study the trafficking of Env to the VS we set out to design a fluorescent protein-tagged Env that is compatible with efficient packaging and viral membrane fusion. To minimize disruption of Env structural stability, we inserted a superfolder allele of GFP [47] directly into the HIV-1 Env coding sequences at selected points of V4 or V5 domain, which have previously been described as producing functional, fusion-competent fluorescent Env [42] (Figure 1A). Four HIV-1 clones carrying the Env-GFP fusion proteins produced 25% to 50% of virus compared to the parent clone, HIV NL4-3 (Figure 1B). Three constructs produced virus with 25 to 50% of infectivity relative to HIV NL4-3 with a wild-type Env (Figure 1C). Western blotting of cells producing HIV-1 Env-V4.1-sfGFP, HIV-1 Env-V4.2-sfGFP and HIV-1 Env-V5.2-sfGFP revealed an expected increase in size of the Env glycoprotein in the cell lysates as compared to WT Env from HIV-1 NL4-3 (Figure 1D). We noted that, in cell lysates, recombinant Env was processed to gp120-GFP fusion, but with a lower efficiency. The recombinant Envs, Env-V4.1-sfGFP, Env-V4.2-sfGFP and Env-V5.2-sfGFP were also packaged efficiently onto virus particles where the processed uncleaved Env on virus particles was observed, but with higher levels of unprocessed gp160 on the virus particle (Figure 1D). One recombinant construct Env-V5.3-sfGFP exhibited higher electrophoretic mobility than the other clones examined, which could be due to impaired intracellular trafficking and glycosylation. This insertion site had previously been associated with slightly lower surface expression in the context of HXB2 Env (80% as compared to WT) yet retained good cell–cell fusion activity [42].

We next examined the efficiency of the four different HIV Env-sfGFP constructs to infect T cell lines. Infection of the highly permissive MT4 cell line was observed with the highest infectivity observed with clone carrying V4.2-sfGFP (Figure 1E). We note that the MT4 cell lines has been well studied as a permissive cell type for certain Env or Gag mutant viruses, which can complement defects that are evident in physiological cell types, such as primary T cells or Jurkat T cells [48,49]. Infection of Jurkat cells was less efficient, with the most the efficient infection by a GFP-Env carrying clone observed by V4.2-sfGFP followed by V4.1-sfGFP and V5.2-sfGFP (Figure 1E). In both MT4 cells and in Jurkat cells, HIV V5.3-sfGFP was non-infectious (Figure 1E). To test if the four HIV clones carrying the Env-GFP fusion proteins can mediate spreading infection, Jurkat cells transfected with each of clones were co-cultured with MT4 cells or Jurkat cells. The spread of virus from transfected donor cells into target cells was measured using flow cytometry (Figure 1F). The infection spread efficiently in MT4 cells with HIV-1 Env-V4.2-sfGFP replicating to a high peak titer as compared to wild type Env construct NL-sfGI, but with slower kinetics. In Jurkat cells infected with a spinoculation method, HIV Env-V4/V5 sfGFP constructs all displayed a lower infection efficiency as compared with wild type Env construct NL-sfGI, and the increase in GFP over time was less sustained over time, relative to MT4 cell infection, indicative of a lower efficiency of spread in these cells (Figure 1G). With an approximately 2-fold increase in infected cells measured between 1-3 days by HIV-1 Env-V4.2-sfGFP, and only a small increase in the percentage of GFP afterwards, this pattern could be explained by the slow spread of the virus, or alternatively by increases in the expression of GFP following an efficient initial spinoculation. To determine if persistence of the HIV-1 Env-V4.2-sfGFP infected cells in culture was due to multiple rounds of infection, we added antiretroviral drugs, before infection or 24 hours post infection. Pre-treatment of cells with 10 μM reverse transcriptase inhibitor AZT or protease inhibitor Nelfinavir blocked viral infection to nearly background levels as measured by Env-GFP from flow cytometry (Figure 1H). Treatment of the infected cultures with anti-retroviral drugs 24 h after infection significantly decreased viral spread compared to non-treated group (Figure 1H, Supplemental Figure S1). Infection was also measured by intracellular p24 staining. Similar to what is observed in Env-GFP by flow cytometry, infection in 24 h anti-retroviral treated groups also showed significantly reduced infection relative to the non-treated condition (Figure 1I). Taken together, these data suggest that HIV Env-V4.2-sfGFP is capable of multi-round infection in Jurkat cells.

### 3.2. Imaging HIV-1 Carrying Fluorescent Env Constructs

**Figure 2:**
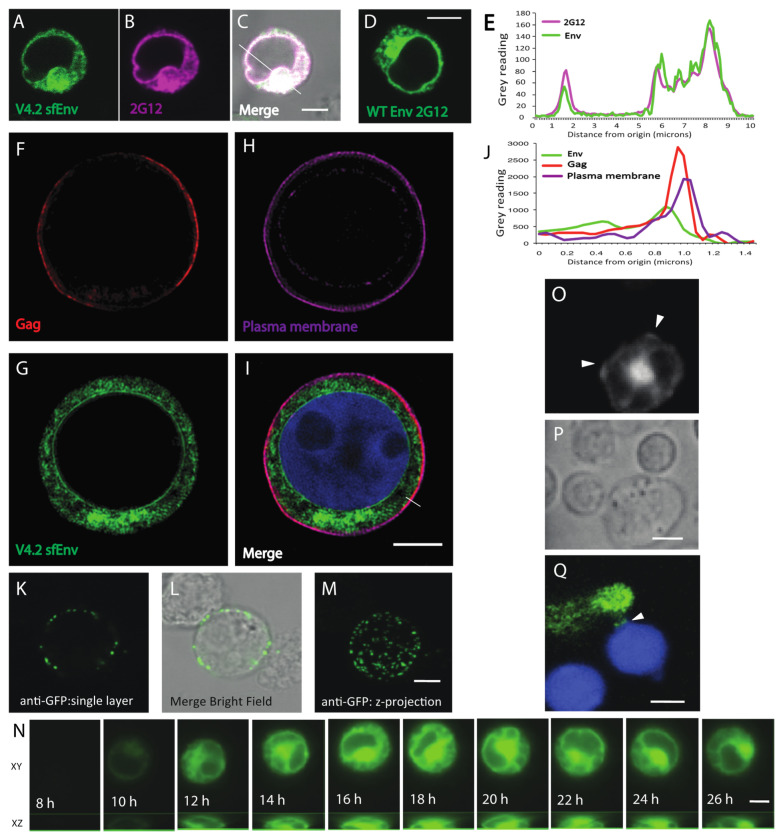
Fluorescence microscopy showing cellular distribution of sfGFP-tagged Env in Jurkat cells. (**A**–**D**) Confocal fluorescence microscopy of Jurkat cells transfected with HIV Env V4.2-Gag-iCherry were fixed and stained with anti-Env mAb 2G12 (Magenta). (**A**) V4.2 sfGFP Env localization, (**B**) 2G12 Env immunostaining, (**C**) Merged image with bright field overlay. (**D**) Confocal microscopy image of WT NL4-3 Env stained with anti-Env mAb 2G12 (Green). (**E**) Graph shows the fluorescence intensity of Env and 2G12 staining traced along the line indicated in (**C**). (**F**–**I**) Super resolution structured illumination imaging of Jurkat cell nucleofected with HIV V4.2-Gag-iCherry were stained with cell mask deep red. (**F**) Cherry-Gag; (**G**), sfGFP-Env, (**H**) Cell Mask; (**I**), Merged image plus nuclear DAPI stain (blue). (**J**) Graph shows the fluorescence intensity of Gag, Env and plasma membrane along the line as indicated in (**I**). (**K**–**M**) Cell surface Env was stained with anti-GFP followed by secondary antibody while cells were alive at 4 °C. The cells were then imaged for surface anti-GFP Env staining, (**K**) single confocal plane; (**L**) single plans merged with bright field; (**M**) Z-projection of stack. (**N**) Example of an HIV V4.2 sfGFP Env nucleofected Jurkat cell was imaged in an Ibidi microchamber slide for over 26 h. Confocal z stacks were acquired at 10-min intervals from 6 h post transfection for 20 h. (**O**) Env-sfGFP fluorescence at site where target cells make contact with a donor Jurkat cell (arrow head-putative synapse). (**P**) Bright field view of (**O**) shows outline of the donor and target cells. (**Q**) Env accumulation at a putative VS formed between an infected primary CD4 T and a target primary CD4 T cell labelled with CellTracker Blue (Blue). Bar: 5 µm.

To study the localization of HIV Gag and Env simultaneously during cell-to-cell spread of HIV-1, we created a series of three dual fluorescent HIV clones carrying a sfGFP fluorescent Env and an mCherry fluorescent Gag. HIV-1 constructs that carry a Cherry fluorescent protein inserted into Gag are not infectious, but generate highly fluorescent virus particles and participate in cell-to-cell transfer [10,46]. We performed immunofluorescence staining of cells infected with HIV-1 Env V4.2 sfGFP-Gag-iCherry, carrying the Env V4.2 sfGFP, the chimeric Env that maintained highest infectivity, to compare the localization of V4.2-sfGFP Env to WT Env. Monoclonal antibody 2G12 binds to a non-conformational epitope and showed colocalization with Env-V4.2-sfGFP fluorescence in a sample cell (Figure 2A–E). V4.2-sfGFP Env is abundantly expressed in cytoplasmic compartments, with the highest fluorescence shown in a peri-nuclear area, consistent with wild type Env distribution reported previously [16]. To assess the distribution of Env and Gag relative to the plasma membrane, we performed structured illumination, super resolution imaging (Deltavision OMXv4.0 BLAZE) of Jurkat cells transfected with HIV-1 Env V4.2 sfGFP-Gag-iCherry and stained with a plasma membrane dye, cell mask deep red (Figure 2F–I). The predominant signal for Env was found in a perinuclear intracellular compartments consistent with an expected localization to the endoplasmic reticulum, Golgi and other endosomal compartments, with comparatively little expression at the cell surface. A line projection of the fluorescence intensity across the plasma membrane revealed that Gag appeared to localize less peripherally compared to the membrane marker. Env was not obviously enriched at the plasma membrane (Figure 2F–J). Surface staining of Env on live cells expressing HIV Env-V4.2-sfGFP with anti-GFP antibody, showed puncta of Env at relatively low density (Figure 2K–M). A time-lapse study of the kinetics of de novo expression of HIV Env-V4.2-sfGFP was performed using a confocal fluorescence imaging system from 6 h to 26 h post transfection (Figure 2N). Env expression in the transfected cells peaked at 16–20 h post transfection (Supplemental Movie S1). To examine the distribution of HIV Env-V4.2-sfGFP during the formation of virological synapses, we co-cultured Env-V4.2-sfGFP transfected Jurkat cells with primary CD4^+^ target cells. Accumulation of Env at the junctions between HIV Env-V4.2-sfGFP transfected Jurkat cells and uninfected primary CD4+ T cells could be observed (Figure 2O,P). In primary CD4^+^ cells transduced with Env-V4.2-sfGFP viruses, synaptic accumulation of Env could be seen at the junction between the HIV-expressing primary T cell and the target primary T cell (Figure 2Q).

### 3.3. A dual Fluorescent Protein-Expressing HIV with Gag-iCherry and Env-sfGFP Participates in VS-Mediated HIV Transfer

**Figure 3:**
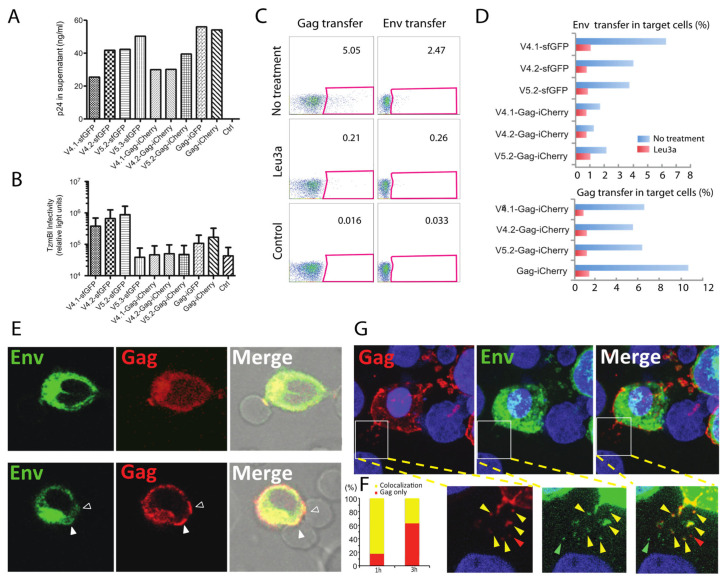
Cell-to-cell HIV-1 transfer assays using dual fluorescent construct of V4/V5-Gag-iCherry. (**A**) Dual fluorescent HIV-1 constructs produce viral particles in 293 T cells as measured by p24 ELISA. Representative example of several transfection experiments is shown. “Ctrl” represents mock-transfected supernatants. In previous studies, HIV-Gag-iGFP and HIV Gag-iCherry produce ~50% WT HIV_NL4-3_ levels of p24 [46]. (**B**) Infectivity of these dual fluorescent HIV-1 constructs using Tzm-bl assay shows infectivity of single fluorescent Env constructs and lack of infectivity of viruses carrying chimeric Gag-iCherry or Gag-iGFP. (**C**) Dual fluorescent constructs HIV-1 V4.2-Gag-iCherry participates in cell-to-cell transfer of HIV from Jurkat to primary CD4 T cells. Flow cytometry measures transfer of Gag-iCherry and Env V4.2-sfGFP signal following cell–cell co-culture, and the transfer is sensitive to CD4 antibody leu3a. Control condition shows background fluorescence of target cells alone. (**D**) Cell-to-cell HIV-1 transfer of Gag and Env measured with indicated fluorescent HIV-1 constructs. (**E**) HIV-1 VS between HIV V4.2-Gag-iCherry transfected Jurkat cells and primary CD4 T cells. Primary CD4 cells from healthy human peripheral blood were co-cultured with transfected donor cells for 3 h. Upper panel: A synaptic button with both Gag and Env is shown between a donor Jurkat cell and a target cell. Lower panel: one donor cell nucleofected with V4.2-Gag-iCherry formed two VSs: the lower synapse shows both Env and Gag concentrated at the cell–cell contact site, while the upper synapse shows Gag accumulation without Env accumulation. (**F**) Analysis of Env and Gag colocalization at virological synapses. Samples fixed at 1 h post co-culture and 3 h post co-culture were compared. VSs, *n* = 17 at 1 h and *n* = 24 at 3 h, counted from 6 fields of view were defined by Gag at the site of cell–cell contact and categorized by reviewers blinded to time point, to enumerate whether Env was colocalized at cell–cell contact site (yellow) or whether Gag was present without Env (red). (**G**) Transfer of both Gag and Env into target cells. Co-cultured cells were fixed and observed by confocal microscopy. Inset shows partial colocalization of transferred Gag and Env. Green, red and yellow arrowheads show Env only, Gag only transfer or co-transfer of both Gag and Env. Bar: 5 µm.

To determine if the fluorescent Env constructs are capable of participating in cell-to-cell HIV transfer across virological synapses, we generated dual fluorescent HIV, which carry two fluorescent protein tags, Cherry and sfGFP, inserted into Gag and Env, respectively. The dual fluorescent viruses make abundant virus particles when transfected (Figure 3A). Similar to the parental HIV Gag-iCherry viruses, the dual fluorescent GFP-Env and Gag-iCherry constructs are non-infectious in reporter cell line TzmBl (Figure 3B). Like HIV Gag-iCherry viruses, these constructs maintained the ability to form VS and transfer Env and Gag into a target cell in a CD4-dependent manner (Figure 3C). HIV V4.2 sfGFP-Gag-iCherry expressing cells were tested for their ability to mediate HIV transfer across VS and transfer of fluorescent Gag and Env was observed (Figure 3C). When the cell co-culture is treated with CD4 antibody, Leu3a, which blocks CD4 engagement with Env, both Gag and Env transfer are blocked (Figure 3C,D). Confocal fluorescence microscopy of the dual fluorescent constructs in Jurkat T cells and primary CD4+ T cells enabled visualization VS where Gag was visualized at the cell–cell junctions with overlapping Env in some cases (Figure 3E, upper panel). In an example of a cell forming two virological synapses, one synapse showed both Gag with some Env signal at the cell–cell junction, and the another shows accumulation of Gag at the cell–cell junction without obvious Env localization (Figure 3E, lower panel). During the imaging of virological synapses, Gag and Env colocalization at a virological synapse was observed soon after cell–cell mixing, and over time, the frequency of VS with only Gag concentrated at the VS appeared to increase (Figure 3F). Both Gag and Env could be observed to transfer into a target cell. Fluorescent HIV proteins transferred into the target cells showed colocalization of Env and Gag at early time points 1 h (Figure 3F), while some puncta appeared to represent the transfer of only Gag or only Env (Figure 3F,G).

### 3.4. Method for Pulse-Chase Labeling of Surface Env Tracks Endocytosis and Relocalization to the VS

**Figure 4:**
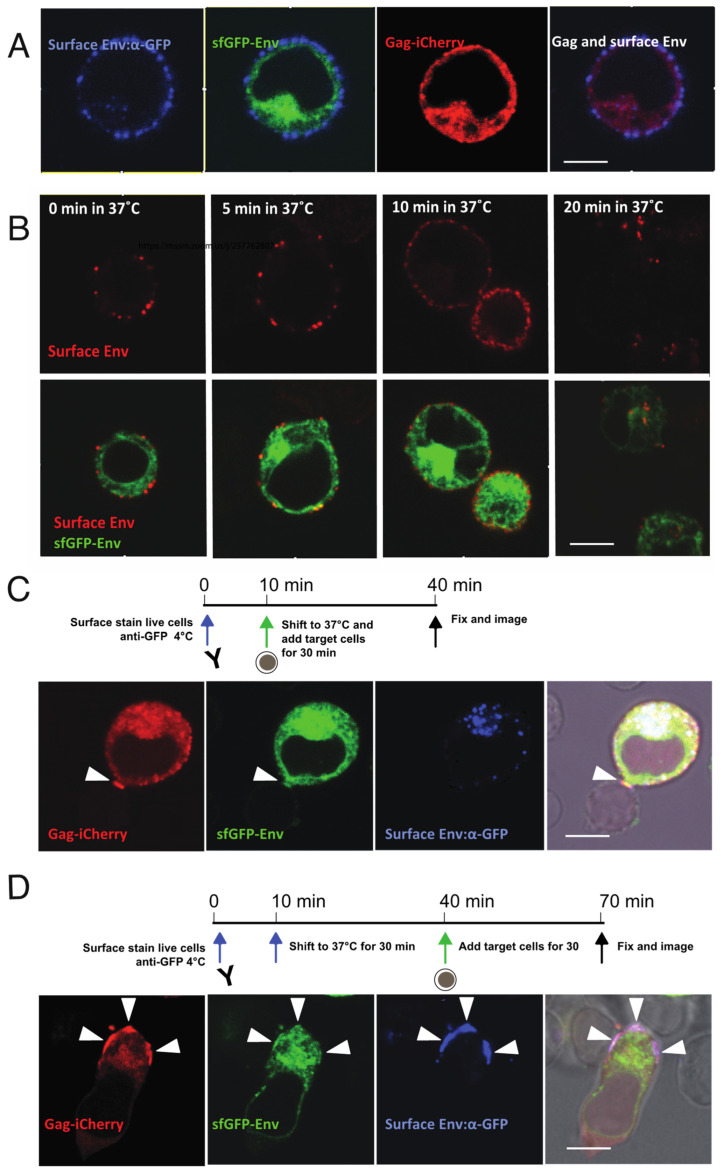
Pulse-chase labeling of cell-surface Env illustrates internalization of Env and relocalization to VS. (**A**) Live cell surface staining of V4.2-Gag-iCherry: nucleofected Jurkat cells stained with anti-GFP antibody at 4 °C. (**B**) Pulse-chase of surface Env to determine time required for endocytosis: cells with surface-stained Env were shifted from 4 °C to 37 °C and kept for indicated time prior to fixation and imaging. (**C**) Stained cell according to the timeline was immediately co-cultured with primary CD4 target cells for 30 min at 37 °C and fixed for imaging. (**D**) Stained cell according to the timeline was first incubated at 37 °C for 30 min, then co-cultured with primary CD4 target cells for another 30 min at 37 °C and fixed for imaging. Arrowheads show VS. Bar: 6 µm.

The Env-CD4 interaction is a prerequisite of T cell-T cell VS formation, but how Env is recruited to the VS is not clear. Given that cell surface GFP Env were not readily apparent, particularly when contrasted with the strong Env signal from intracellular sites (Figure 2G), we examined methods to stain and track cell surface pools of Env to follow the fate of the fraction of Env that is at the plasma membrane. To examine the pathway of Env recruitment, we developed a surface Env labeling protocol with an anti-GFP fluorophore conjugated antibody and performed a pulse-chase imaging study to follow movements of surface-localized Env over time. Cell surface Env of a Jurkat cell nucleofected with HIV-1 V4.2-Gag-iCherry was visualized by staining at 4 °C (Figure 4A). We examined surface Env stained cells over time to examine the rate at which Env is endocytosed from the cell surface [13]. After warming cells to 37 °C, cells were fixed after 5, 10 and 20 min to monitor the movement of pulse labeled Env (Figure 4B). Env progressively moves from external to internal compartments over this time frame. The surface Env stained cells were separated into two groups: one group that was mixed with target cells immediately after surface staining, and co-cultured at 37 °C for 30 min (Figure 4C). The second group was allowed to recover at 37 °C for 30 min, then mixed with target cells for another 30 min (Figure 4D). Both groups of cells were fixed afterwards and imaged with confocal microscopy. In group 1, surface labeled Env was mainly found in endocytic compartments (as defined by endocytosed Ab label), while at the synapse area, no labeled Env was observed (Figure 4C). In the second group, which was allowed to recover prior to cell–cell mixing, which provided time for Env endocytosis, Env was observed at the cell–cell junction at a newly formed VS (Figure 4D). These qualitative imaging studies indicate that surface-labeled Env can be endocytosed, and can also subsequently be observed to localize to the VS.

### 3.5. Fluorescence Recovery after Photobleaching (FRAP) of HIV Env V4.2 sfGFP-Gag-iCherry at VS Reveals Constitutive Turnover of Env near the VS

**Figure 5:**
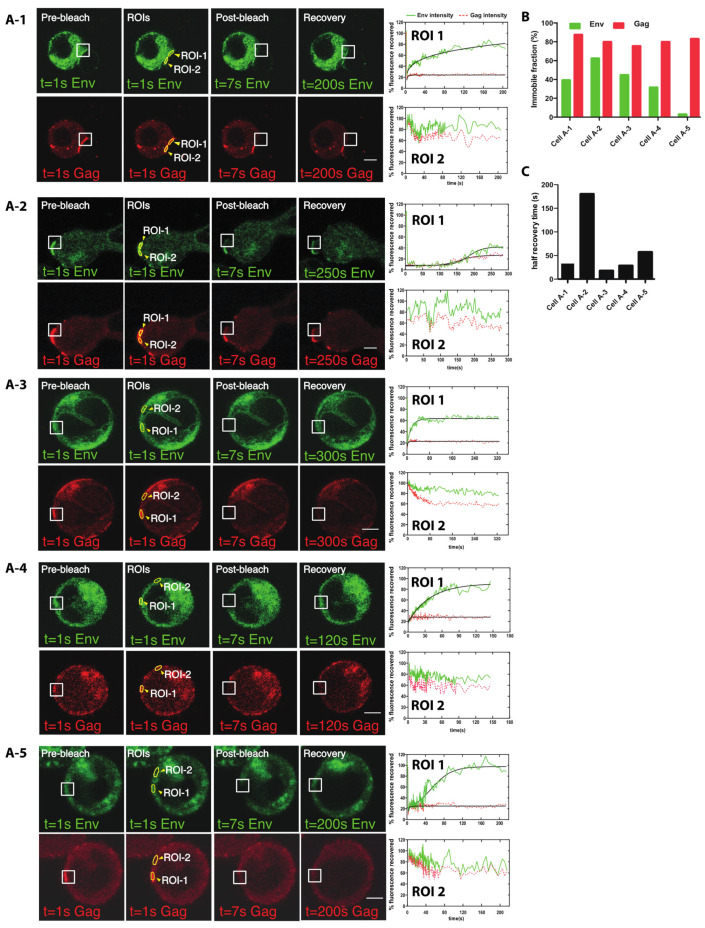
Rapid Env fluorescence recovery after photobleaching was observed at the VS. CD4+ T cell line Jurkat was nucleofected with HIV Env V4.2 sfGFP-Gag-iCherry and mixed with primary CD4 T cells and allowed to form VSs. Established synapses where Gag and some level of Env was observed at cell–cell contacts were selected for FRAP imaging. (**A-1**–**A-5**) Before photobleaching (left panels) a virological synapse with both Gag and Env could be observed between a donor cell and a target cell. A region covering part of the synaptic junction is bleached as marked by the white square. After photobleaching, fluorescent recovery measured in Env and Gag in selected regions of interest (ROI). ROIs were selected on bleached synapse or an unbleached area as shown in closed yellow region. A fluorescence intensity curve describing the fluorescence recovery is shown (left). (**B**) Immobile fraction of each FRAP experiment is indicated. (**C**) Graph shows the t half recovery time of each cell (**A-1**–**A-5**). Bar: 3 µm.

How Gag recruitment may influence Env at the VS is not known. It is possible that the recruitment of Gag to the VS may trap Env during its incorporation onto nascent virus particles, or that the interaction of Env with CD4 may immobilize it at the cell surface. To simultaneously track the kinetics of Env and Gag recruitment to the VS, we performed fluorescence recovery after photobleaching (FRAP) experiments with HIV V4.2 Env sfGFP-Gag-iCherry to measure the rate of turnover of Env and Gag at VS. We identified cells with a VS that showed both Gag and Env colocalized at the cell contact area. Half of the VS was photo-bleached, and the other half of the VS left unbleached, to allow segmentation of the VS and measurement of recovered fluorescence over time. Additional unbleached areas were tracked over time as a control to determine the basal rate of photodecay. As shown in Figure 5A-1, the white square indicates the bleached area, and the yellow closed region is the selected region of interest (ROI). ROI-1 is the bleached synapse area, while ROI-2 is the unbleached control area. A steady recovery of Env intensity was observed within about 200 s, while, in the same time period, there was minimal fluorescence recovery of Gag (Supplemental Movie S2). Four additional FRAP studies on four different virological synapses were performed (Figure 5A-2 to Figure 5A-5, see Supplemental Movies S2–S6). The recovery curve of Env was fitted to a one-phase exponential association function for each ROI (Figure 5A-1 to Figure 5A-5, right panels, ROI curves). The Env intensity before bleaching was set to 100%. The maximum recovery over the time frame of imaging was used to calculate an immobile fraction which differed between the different samples (Figure 5B). In all the VS we observed, Gag fluorescence recovery was not observed, while Env fluorescence recovery occurred within 2-3 min with the half recovery time (Figure 5C), indicating a much greater rate of Env turnover at the VS relative to Gag.

### 3.6. High Turnover of Env Proximal to the VS Requires Endocytosis of Env Using a Membrane Proximal Tyrosine Y712

**Figure 6:**
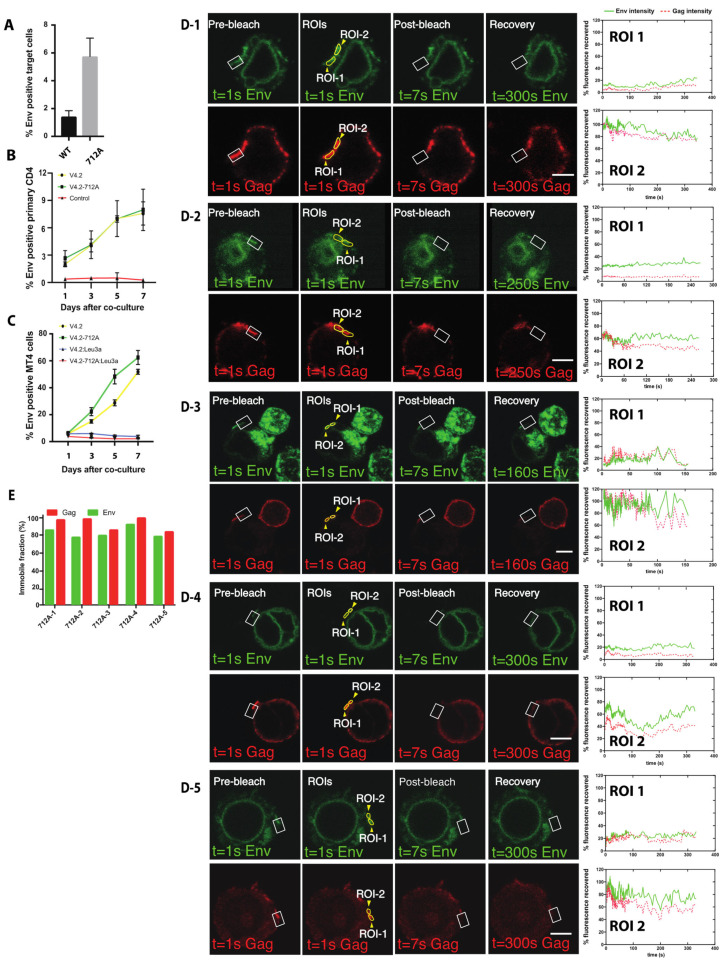
Env fluorescence after photobleaching does not recover when examining Y712A mutants of Env V4.2-sfGFP in FRAP. (**A**) Jurkat cells nucleofected with wild type Env-V4.2-sfGFP or Env-V4.2-Y712A-sfGFP were co-cultured with primary CD4 cells for 3 h. Env transfer to primary CD4 cells were determined by Flow cytometry. (**B**) Jurkat cells nucleofected with wild type, Env-V4.2-sfGFP, or Env-V4.2-Y712A-sfGFP were co-cultured with activated primary CD4 cells to monitor productive infection in target cells. Samples were collected on day 1, 3, 5, 7 to determine the portion of primary CD4 cells with fluorescent Env. (**C**) Jurkat cells nucleofected with wild type, Env-V4.2-sfGFP, or Env-V4.2-Y712A-sfGFP were co-cultured with MT4 cells for days to monitor productive infection in target cells. Samples were collected on day 1, 3, 5, 7 to determine the portion of MT4 cells with fluorescent Env. (**D**) Fluorescence recovery after photobleaching (FRAP) of Env and Gag virological synapse with HIV V4.2-712A-Gag-iCherry. Before photobleaching a VS, both Gag and Env are concentrated at the junction between a donor cell and a target cell. A region of interest covering part of the synaptic button was bleached as shown in white square. ROIs were selected on bleached synapse (ROI-1) or an unbleached area (ROI-2) as shown in closed yellow region. Recovery curves of five individual experiments are displayed in (**D-1**–**D-5**). (**E**) shows the immobile fraction of each FRAP experiment. Bar: 5 µm.

The gp41 C-terminal membrane-proximal tyrosine 712 in a YXXL AP-2 binding-motif is important for the internalization of surface Env through AP-2 mediated endocytosis [23]. To test if surface Env endocytosis is required for synapse recruitment or turnover of Env at the VS, we introduced the Y712A point mutation into the viral clone, HIV Env-V4.2-sfGFP. HIV-1 with the Env Y712A mutation is reported to be less infectious as compared to wild type virus [44]. We performed a T cell-to-T cell viral transfer assay using Jurkat donor cells and primary CD4 T cells as target cells and observed that cell-to-cell transfer of Env is increased by 3-fold in Y712A mutant relative to non-mutated virus in 3-h co-culture (Figure 6A). A separate cell-to-cell infection assay was performed to measure productive infection between HIV-expressing Jurkat cells and primary CD4 T cells. In this assay, both the 712-wild type (WT) and the Y712A virus spread with similar efficiencies (Figure 6B). In highly permissive MT4 cells, the Y712A virus spread with a slightly higher rate than wild type in 7-day productive infection (Figure 6C). We next performed live imaging to see if the mutation which disrupts Env endocytosis from cell surface permits VS formation and accumulations of Env and Gag at the cell–cell junctions. We readily observed VS formation with high levels of Env recruitment to the synapse with the Y712A mutation. When conducting FRAP studies we found that the Env recovery was dramatically decreased in the HIV-1 Env V4.2-Y712A-sfGFP when compared to the non-mutated clone (Figure 6D-1, Supplemental Movie S7). Notably the distribution of Env Y712A-sfGFP within intracellular compartments showed prominent circumferential perinuclear staining, and lesser staining of a larger perinuclear compartment, perhaps indicative of the loss of endocytic pools of Env. Four additional FRAP experiments were performed on virological synapses formed by HIV-V4.2-Y712A-sfGFP (Figure 6D-2 to Figure 6D-5). There was minimal or no recovery of Env or Gag observed over 5 min after photobleaching. Videos of all five virological synapses are in Supplemental Movies S7–S11. Based on the extent of the fluorescence recovery, the immobile fraction of Env was calculated, which was close to 100% in all the examples (Figure 6E).

## 4. Discussion

In this study, we have constructed a fluorescent Env-carrying HIV clone that is still capable of viral entry and productive infection in T cells in cell culture. The fluorescent Env-GFP fusion protein resembles wild type Env in its subcellular distribution and is still able to participate in VS formation and cell-to-cell infection. By utilizing a superfolder allele of GFP and employing favorable insertion sites described by Nakane et al. into molecular clone NL4-3, we provide a description of an HIV-1 clone encoding a fluorescent Env that is autonomously infectious, although with attenuated infectivity. This tool makes it possible to observe Env distribution and trafficking within the context of productive infections, and in the absence of helper virus. We employ it here to test models for how Env trafficking contributes to viral spread between cells and supports the production of infectious virus particles.

Immunofluorescence with monoclonal antibody, 2G12, which recognizes a carbohydrate epitope, revealed that the localization of V4.2 Env resembles native HIV-1 Env. The localization of Env we observed is consistent with the results of previous studies of Env-GFP fusion protein by Nakane et al., produced outside the context of a molecular clone of HIV [42]. With super-resolution imaging and surface Env staining, we observed that the majority of Env is expressed in internal compartments, with localization compatible with expected biosynthetic pathway of Env in the endoplasmic reticulum, the Golgi apparatus, and endosomal compartments. As previously appreciated, cell surface Env represents a small fraction of total Env in the cell, and the results of our fluorescence microscopy also show very low surface Env levels [50,51,52], which also appears to correlate with the low Env density on viral particles (7–14 Env trimer/particle) [53,54].

When imaging VS, the fluorescent Env construct could identify increased concentrations of surface-targeted Env at cell–cell contact zones. This localization is consistent with the earliest VS imaging studies on fixed samples that found that Env accumulates to the VS area through actin-dependent processes [3]. In the studies presented here, cell surface Env was a small fraction of the total cell fluorescence and was best discriminated when detected with fluorescent secondary antibodies that stained only surface Env. When visualized with GFP alone, V4.2 Env density at the cell surface was relatively sparse and evenly distributed, with no obvious areas where Env is pre-accumulated prior to VS formation (Figure 2K–M and Figure 4A).

The cell surface Env distribution before and after VS formation exhibits two different patterns: diffuse versus focal. The initial broad distribution of Env on the cell surface occurs prior to target cell engagement, and retargeting of the recycled Env to the VS appears to occur following CD4 engagement and may facilitate efficient particle incorporation. Evidence of VS-targeted Env trafficking can be observed prior to accumulation of Gag at the VS. When an infected cell is attached to an uninfected target cell, Env accumulation can be observed within minutes after cell attachment [39]. The post synaptic polarized targeting of Env to the VS displays many functional similarities to secretory pathways that can be polarized when cells are engaged in immunological synapses [55,56,57].

To explore the relationship of Gag and Env during the formation of VSs, a dual-fluorescent virus carrying Gag-iCherry and Env-V4/V5-isfGFP fusion proteins was studied. Although the clone is non-infectious, the dual fluorescent construct can also efficiently engage in cell-to-cell transfer of HIV-1, which occurred in a CD4 dependent manner. The ability to mediate cell-to-cell HIV transfer indicates that the CD4 binding sites of these constructs are functional, and signaling events prior to and during VS formation are likely to be intact. Using this construct, a surface labeled Env pulse-chase protocol suggested that the display of Env on cell surface is followed by internalization and subsequent concentration at the VS. A limitation of the current studies is that the use of the dual fluorescent clone is itself not infectious, due to a defect mediated by the Gag-iCherry insertion, although our previous studies using these constructs provides evidence that transfer of Gag-iCherry particles, resembles immature WT particles when examined by electron microscopy [38,46]. Given that the iGFP insertion or iCherry insertion is not wild type and is non-infectious, it is plausible that the GFP insertion may impact issues of Gag and Env turnover, while maintaining CD4-dependent viral transfer.

Early confocal imaging studies revealed the VS as a site where button-shaped accumulation of Gag formed at the adhesive junction between an infected cell and a target cell [40]. Electron microscopy of the virological synapse revealed Gag accumulation in electron dense crescents forming a tight lattice at the VS [38,40]. Recruitment of Gag to the VS occurs from the lateral migration of plasma membrane-targeted Gag that moves towards the site of cell–cell contact site over minutes [40]. FRAP studies here show that, at a late stage of VS formation, after Gag synaptic button is established, Gag is largely immobile, and shows limited recovery after photobleaching at the VS. This consistent with a largely irreversible incorporation of Gag into nascent budding particles [58]. Compared to Gag, Env can also be observed at or near the cell–cell contact area but at a lower relative fluorescence signal (Figure 3F). A proposed model for Env incorporation into a budding virus particle is that it may be mediated by “trapping” of Env with its long cytoplasmic tail becoming encumbered in the 2-dimensional Gag lattice [28]. However, in contrast to Gag at the VS, which is not exchanging with other pools of Gag in the cell, a large fraction of Env continues to exchange with intracellular pools even after stable VS formation. This ability to exchange freely may indicate that some fraction of Env is incorporated after Gag accumulates at a late stage of assembly, where it may still exchange with areas in the vicinity of the target cell membrane. This could be consistent with a recent superresolution imaging study suggests that Env is packaged at a late-stage of assembly and is localized with a distribution biased toward the necks of budding viruses [59].

In our FRAP studies (Figure 5), a majority of Env at the bleached area recovered within minutes of photobleaching, resulting in different final immobile fractions for Env. This indicates the while a large fraction of Env is continuously recycling to the VS a relatively smaller, variable fraction can be immobilized at the VS. The state of the cell, the stage of VS formation and the size of the VS all may contribute to these differences in the immobile fraction. The biosynthesis of HIV Env and Gag occurs through different pathways. In this paper, our FRAP studies indicate that the forces that maintain Gag and Env at the VS are distinct. If there are stable Env Gag complexes at the VS, the levels of Env retained represent a minor fraction of the protein at the VS. The high degree of recovery after FRAP also indicate that a majority of Env is not immobilized in the vicinity of the VS by other interactions, for example by the interaction of Env with CD4 on the target cell. We note, however, that it likely takes a very small amount of Env to initiate a VS, so this does not rule out a role for an immobilized fraction of Env at the VS.

We characterized an endocytic Env mutant and performed FRAP at the VS and observed that Env could still accumulate at the VS; however, the recycling of Env to the VS was not observed. This shows that blocking the endocytosis of Env with a Y712A mutation abolishes the turnover of Env at the VS. In this case, the accumulation of Y712A Env at the VS may be driven by the high concentration of Env at the cell surface. Truncation mutants in the C-terminal tail of Env or elimination of the main endocytic motif, Y712, allow high levels of Env to be displayed on cell surface [20,25]. An intact cytoplasmic tail is required for incorporation into the “neck” of the emerging budding virus and it is suggested that Env that are missing the CT are passively incorporated into viral particles [59]. In our experiments, the Y712A endocytosis mutant leads to more viral transfer through the VS, though shows a modest impact on the overall infectivity. This mutant can display different phenotypes depending upon the cell line it is tested in, though, in general, it is still infectious [60]. Together these data indicate that recycling is dispensable for VS formation, transfer and infection. We therefore speculate that a major role of recycling of Env at the VS lies in immune evasion: keeping surface Env density low to escape from immune surveillance [61,62]. Other studies from our group have shown Y712A mutants can also impact Env cell surface conformation and modulate the ability of broadly neutralizing monoclonal antibodies to neutralize cell-to-cell infection [63].

**Figure 7:**
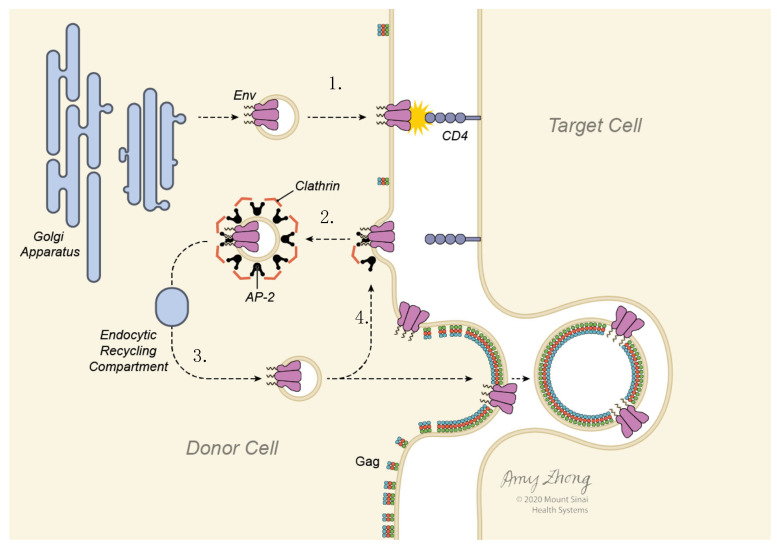
Model of Env trafficking pathways that support Env accumulation at the VS. (**1**) Env is transported to the cell surface following synthesis through the ER/Golgi pathways. (**2**) Clathrin-mediated endocytosis is initiated by recognition of the Env cytoplasmic tail by adapter protein complex, AP-2, which recognizes the membrane proximal tyrosine motif in Env. (**3**) Following internalization Env is recycled back to the cell surface, selectively trafficking to the VS where it can be incorporated into nascent virus particles. (**4**) Env at the VS continues to recycle while Gag does not exchange.

In summary, these imaging studies support an emerging model of HIV-1 cell-to-cell infection, where Env traffics between the cell surface and endocytic compartments before being packaged onto a budding virus particle (Figure 7). An initial transient phase of exposure at the cell surface participates in the detection of the target cell. Subsequently Env that is recycled from surface, is redirected specifically to the VS (through an unknown mechanism), where Env is incorporated into virus. Env trafficking to VS can produce higher steady state concentrations of this protein at the cell–cell contact areas, regardless of whether Env is recycled. The process of recruitment to the VS is therefore optimized to promote efficient transfer of virus from cell to cell while maintaining minimal surface expression of the dominant viral surface antigen.

## Data Availability

Not applicable

## Supplementary Materials

The following are available online at https://www.mdpi.com/article/10.3390/v14010038/s1, Supplemental Figure S1: Flow cytometry plots from Figure 1H,I. Movie S1: Time lapse confocal images of a Jurkat cell after nucleofection with HIV Env V4.2 sfGFP and monitored in an Ibidi microchamber slide from 8 to 26 h post transfection. Movie S2: FRAP of Env and Gag at the virological synapse with HIV V4.2-712A-Gag-iCherry, cell from Figure 5A1. Before photobleaching a VS, both Gag and Env are concentrated at the junction between a donor cell and a target cell. A portion of the VS indicated by a white square was bleached and the recovery of fluorescence to the bleach site monitored over time. Movie S3: FRAP movie of Gag and Env at a VS with WT Env, cell from Figure 5A2. Movie S4: FRAP movie of Gag and Env at a VS with WT Env, cell from Figure 5A3. Movie S5: FRAP movie of Gag and Env at a VS with WT Env, cell from Figure 5A4. Movie S6: FRAP movie of Gag and Env at a VS with WT Env, cell from Figure 5A5. Movie S7: FRAP movie of Gag and Env at a VS with Mutant Env Y712A, cell from Figure 6D1. Movie S8: FRAP movie of Gag and Env at a VS with Mutant Env Y712A, cell from Figure 6D2. Movie S9: FRAP movie of Gag and Env at a VS with Mutant Env Y712A, cell from Figure 6D3. Movie S10: FRAP movie of Gag and Env at a VS with Mutant Env Y712A, cell from Figure 6D4. Movie S11: FRAP movie of Gag and Env at a VS with Mutant Env Y712A, cell from Figure 6D5.
