# Supplementary material for: A Replication-Competent HIV Clone Carrying GFP-Env Reveals Rapid Env Recycling at the HIV-1 T Cell Virological Synapse
Source: Viruses. 2021 Dec 25;14(1):38. doi: 10.3390/v14010038 (PMC8781834; doi:10.3390/v14010038)
Supplement: Supplementary file 1 [file viruses-14-00038-s001.zip › Supplemental Figure S1.pdf]

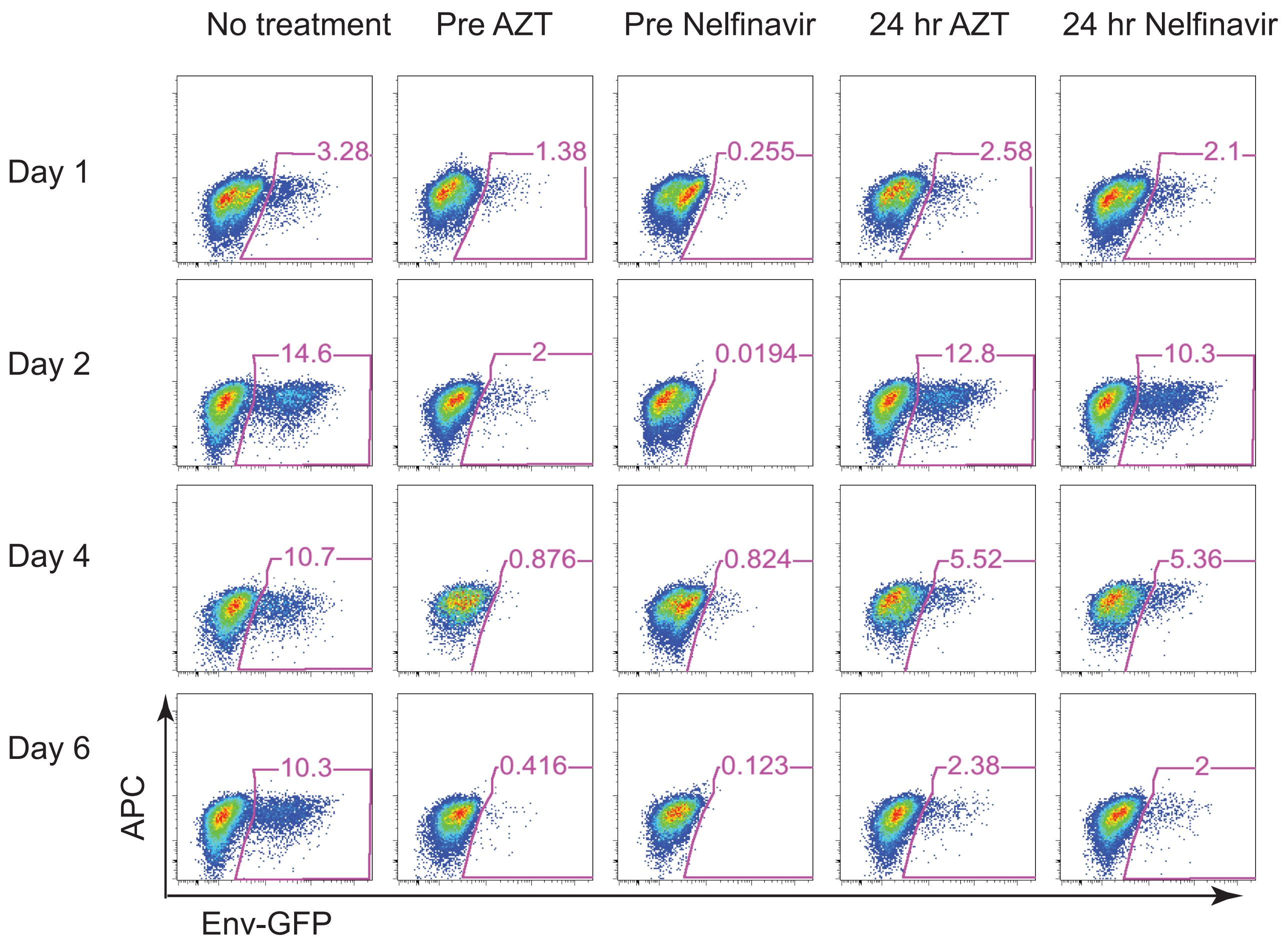

Supplemental Figure S1. Growth curve of HIV Env-V4.2-sfGFP in Jurkat cells with and without of anti-retroviral treatment. Jurkat cells were treated with or without 10μM AZT and/ or Nelfinavir prior to or 24 hr after spinoculation. Samples were collected on day 1, 2, 4 and 6 for flow cytometric analysis of Env-GFP fluorscence signal.
